# Magnitude and influencing factors of respiration-induced liver motion during abdominal compression in patients with intrahepatic tumors

**DOI:** 10.1186/s13014-016-0762-z

**Published:** 2017-01-10

**Authors:** Yong Hu, Yong-Kang Zhou, Yi-Xing Chen, Zhao-Chong Zeng

**Affiliations:** Department of Radiation Oncology, Zhongshan Hospital, Fudan University, 180, Feng Lin Road, Shanghai, 200032 China

**Keywords:** Four-dimensional computed tomography, Abdominal compression, Body mass index (BMI), Respiratory liver motion

## Abstract

**Purpose:**

The purpose of this study was to use 4-dimensional-computed tomography (4D-CT) to evaluate respiration-induced liver motion magnitude and influencing factors in patients with intrahepatic tumors undergoing abdominal compression.

**Methods:**

From January 2012 to April 2016, 99 patients with intrahepatic tumors were included in this study. They all underwent 4D-CT to assess respiratory liver motion. This was performed during abdominal compression in 53 patients and during free-breathing (no abdominal compression) in 46 patients. We defined abdominal compression as being effective in managing the breath amplitude if respiration-induced liver motion in the cranial-caudal (CC) direction during compression was ≤5 mm and as being ineffective if >5 mm of motion was observed. Gender, age, body mass index (BMI), transarterial chemoembolization history, liver resection history, tumor area, tumor number, and tumor size (diameter) were determined. Multivariate logistic regression analysis was used to analyze influencing factors associated with a breath amplitude ≤5 mm in the CC direction.

**Results:**

The mean respiration-induced liver motion during abdominal compression in the left-right (LR), CC, anterior-posterior (AP), and 3-dimensional vector directions was 2.9 ± 1.2 mm, 5.3 ± 2.2 mm, 2.3 ± 1.1 mm and 6.7 ± 2.1 mm, respectively. Univariate analysis indicated that gender and BMI significantly affected abdominal compression effectiveness (both *p* < 0.05). Multivariate analysis confirmed these two factors as significant predictors of effective abdominal compression: gender (*p* = 0.030) and BMI (*p* = 0.006). There was a strong correlation between gender and compression effectiveness (odds ratio [OR] = 7.450) and an even stronger correlation between BMI and compression effectiveness (OR = 10.842).

**Conclusions:**

The magnitude of respiration-induced liver motion of patients with intrahepatic carcinoma undergoing abdominal compression is affected by gender and BMI, with abdominal compression being less effective in men and overweight patients.

## Introduction

Liver cancer is much more common in men than in women. In men, it is the second leading cause of cancer death worldwide and in less developed countries. In more developed countries, it is the sixth leading cause of cancer death among men. An estimated 782,500 new liver cancer cases and 745,500 deaths occurred worldwide during 2012, with China alone accounting for about 50% of the total number of cases and deaths [1].

Patients with unresectable but limited hepatocellular carcinoma (HCC) recurrence may undergo external-beam radiation therapy (EBRT), but hepatic tumors move during EBRT because of respiration-induced liver motion. In order to avoid both inadequate tumor coverage and unnecessary liver parenchyma irradiation, it is crucial to determine the internal target volume (ITV). Abdominal compression (AC) can be used in conjunction with 4-dimensional computed tomography (4D-CT) to reduce liver respiratory motion and determine the ITV [2]. Mid-ventilation is an attractive strategy because it allows smaller planning target volume (PTV) margins to account for breathing motion [3]. It seems not crucial for radiation oncologists to determine the ITV for patients when using breath-hold techniques, gated treatment, or tracking techniques, all of which have already eliminated the influence of breathing motion, but the reproducibility and accuracy of these techniques should be included in the PTV margin [3, 4].

The ITV boundary range relies primarily on respiration-induced liver motion, and if not properly accounted for, motion of this magnitude could lead to altered dosimetry because of the use of a static plan and irradiation of an uncertain volume of normal tissue [5, 6]. Smaller target volumes can improve dose distribution in normal liver tissue and provide better target dose coverage [7]. Concern about toxicity to normal tissue can be partially addressed by improving the geometrical targeting accuracy and confidently reducing treatment margins [8]. Therefore, it is imperative to manage and/or account for respiratory liver motion.

AC is commonly used for reducing abdominal tumor motion during radiation therapy [9, 10]. In previous studies, dosimetric comparison research of liver tumor radiotherapy was mainly based on the 5 mm expansion that was added to the gross tumor volume to create the PTV [7, 11, 12]. Lujan et al. [13] also reported that static dose distributions would change significantly when the amplitude of motion was more than 5 mm. Respiration-induced liver motion is anisotropic, occurring primarily in the cranial-caudal (CC) direction [14–18]. Based on the above observations, we consider AC to be effective if respiration-induced liver motion is maintained within 5 mm in the CC direction.

In the current study, we used 4D-CT scans to investigate the magnitude of the reduction of respiration-induced liver motion achieved and to identify the influencing factors that would help predict the effectiveness of AC for patients with intrahepatic tumors.

## Materials and methods

### Patients

The patient inclusion criteria were as follows: (1) confirmed liver hepatic malignancy and plan to receive EBRT; (2) presence of at least one hepatic tumor; (3) Child-Pugh A liver function and Karnofsky performance status > 80; (4) no colostomy or ascites; (5) no history of chest surgery; (6) regular breathing after basic breath training; (7) no disease affecting pulmonary function. (8) AC of the subxiphoid area was possible; and (9) maximum compression force could be reached.

Between January 2012 and April 2016, 53 consecutive patients (41 male and 12 female; age range 18–82 years; 46 primary liver cancers and 7 metastatic liver cancers) diagnosed with liver cancer were included in the study and underwent 4D-CT scans to assess respiratory liver motion with AC. Another 46 patients with intrahepatic carcinoma (32 male and 14 female; age range 40–81 years; 40 primary liver cancers and 6 metastatic liver cancers) were also included and underwent 4D-CT scans to assess respiratory liver motion without AC.

### Abdominal compression

All patients received AC using the Body Pro-Lok system (CIVCO, Orange City, IA, USA), which consisted of a lightweight carbon fiber platform, a patient customizable vacuum cushion, an AC bridge, a respiratory plate, and knee and foot sponges. Each patient underwent basic respiratory training guided by a radiotherapy oncologist and therapist before administration of AC. AC was applied during each patient’s end-expiration until maximum tolerability was reached, as indicated by the patient. The AC was applied to the subxiphoid area.

### 4D-CT image acquisition

4D-CT scans were obtained using a CT-simulation Scanner (Siemens Somatom CT, Sensation Open; Siemens Healthcare, Munchen, Germany). Patients were placed in the supine position with their arms raised above the forehead and were immobilized using a vacuum cushion. Patient respiration was detected using the Respiratory Gating System (AZ-733 V, Anzai Medical, Tokyo, Japan). The x-ray tube settings were as follows: 120 kV; 400 mA; pitch 0.1; 3-mm reconstructed thickness; and gantry rotation cycle time 0.5 s for patients without AC when the respiratory cycle of each patient was ≤5 seconds, and 1 s for patients under AC when the respiratory cycle of each patient was >5 s to avoid 4DCT image quality reduction and reconstruction distortion. The respiratory phase on the respiratory wave was manually adjusted and confirmed by the CT-simulation technician prior to CT image reconstruction. 4D-CT images from raw respiratory data were sorted into a 10 CT image series (CT0, CT10…CT90) according to the respiratory cycle, with CT0 being defined as the end-inspiration phase and CT50 as the end-expiration phase [19]. Datasets for 4D-CT scans were then transferred to Nucletron Oncentra’s treatment planning software Version 4.3(NUCLETRON B.V., Veenendaal, Netherlands), and all liver contours were drawn by an experienced observer (HY) and confirmed by a single physician (YKZ).

### Liver displacement acquisition and analysis

Liver contours were delineated at all CT image phases and then copied manually to a single plan. The nine liver contours of CT10, CT20…CT90 were copied onto the CT0 image and were designated Copy_Contour10_, Copy_Contour20_…Copy_Contour90_. There were 10 liver contours (Copy_Contour10_, Copy_Contour20_…Copy_Contour90_ and liver contours of CT0) on the CT0 image. Then, 0- and 90° digitally reconstructed radiographic beams were added to the CT0 image. 0- and 90° digitally reconstructed radiographic images were a set of coronal and sagittal projections. Ten liver 3-dimensional (3D) contours could be projected onto the digitally reconstructed radiographic images in the directions of 0 and 90°. Overlays of 10 liver contours were shown on the digitally reconstructed radiographic images of 0 and 90°. The relative coordinates of the liver were automatically generated to calculate the respiratory liver motion in three different anatomical directions. The position for each liver was expressed using the left-right (LR), CC, and anterior-posterior (AP) coordinates of the center of mass (COM) for each 4D-CT bin. Then, the range in respiratory liver motion from the COM of each coordinate was obtained. Maximum range of motion in each axial direction was calculated by subtracting the minimum relative coordinate value from the maximum relative coordinate value.

In this study, we defined that the AC is just effective if respiration-induced liver motion is less than 5 mm in CC direction.

### Formulas

Liver motion was also expressed as a 3D vector, which was calculated as the quadratic mean of the motions in three orthogonal directions according to the following formula:

$$ \mathrm{V}={\left({\Delta \mathrm{L}\mathrm{R}}^2+{\Delta \mathrm{C}\mathrm{C}}^2+{\Delta \mathrm{A}\mathrm{P}}^2\right)}^{1/2} $$

Body mass index (BMI) was calculated using weight (kg) divided by the square of the height (m), according to the following formula:

$$ \mathrm{B}\mathrm{M}\mathrm{I}=\mathrm{weight}/{\mathrm{height}}^2 $$

### Statistical analyses

Variations in the LR, CC, AP, and 3D directions are expressed as mean ± standard deviation. The Chi-square test was used for univariate analyses (Table 1). Multivariate logistic regression analysis was used to analyze the influencing factors associated with breath amplitude (Table 2). The independent-samples *t*-test was used to compare differences in male and female mean BMI values, and differences in liver respiratory motion in the CC direction between male and female patients without AC. Pearson correlation analysis was used to detect the correlation between free-breathing amplitude in the CC direction and BMI for patients without AC. All calculations were performed using SPSS 15.0 for Windows (Chicago, Illinois, USA). For all statistical tests, the *p*-value for significance was set at < 0.05.

**Table 1:** Univariate analyses of factors associated with effectiveness of abdominal compression

Clinicopathological factors	Breath amplitude in CC direction		*p*-value
	≤5 mm	>5 mm	
Gender, n (%)			
Male	17 (41.5%)	24 (58.5%)	0.041*
Female	9 (75.0%)	3 (25.0%)	
Age, n (%)			
≤ 50 y	11 (55.0%)	9 (45.0%)	0.500
> 50 y	15 (45.5%)	18 (54.5%)	
BMI, n (%)			
< 25 kg/m^2^	23 (62.2%)	14 (37.8%)	0.004*
≥ 25 kg/m^2^	3 (18.7%)	13 (81.3%)	
TACE, n (%)			
Yes	15 (51.7%)	14 (48.3%)	0.669
No	11 (45.8%)	13 (54.2%)	
Postoperative recurrence, n (%)			
Yes	10 (45.5%)	12 (54.5%)	0.659
No	16 (51.6%)	15 (48.4%)	
Liver tumor location, n (%)			
Right lobe	18 (50.0%)	18 (50.0%)	0.691
Left lobe	2 (33.3%)	4 (66.7%)	
Left and right lobe	6 (54.5%)	5 (45.5%)	
Intrahepatic lesions, n (%)			
Solitary	15 (48.4%)	16 (51.6%)	0.908
Multiple	11 (50.0%)	11 (50.0%)	
Tumor diameter, n (%)			
≤ 5 cm	19 (48.7%)	20 (51.3%)	0.934
> 5 cm	7 (50.0%)	7 (50.0%)	

*Abbreviations*: *BMI* body mass index, *CC* cranial-caudal, *TACE* transarterial chemoembolization. * statistically significant values

**Table 2:** Multivariate logistic regression analyses of factors associated with effectiveness of abdominal compression

Parameter	Multivariate Analysis		
	OR	95% CI	*p*-value
Gender			
Male	1		0.030*
Female	7.450	1.221–45.473	
BMI			
≥ 25 kg/m^2^	1		0.006*
< 25 kg/m^2^	10.842	2.012–58.434	

*Abbreviations*: *BMI* body mass index, *CI* confidence interval, *OR* odds ratio. * statistically significant values

## Results

### Respiratory liver motion during abdominal compression

**Fig. 1:**
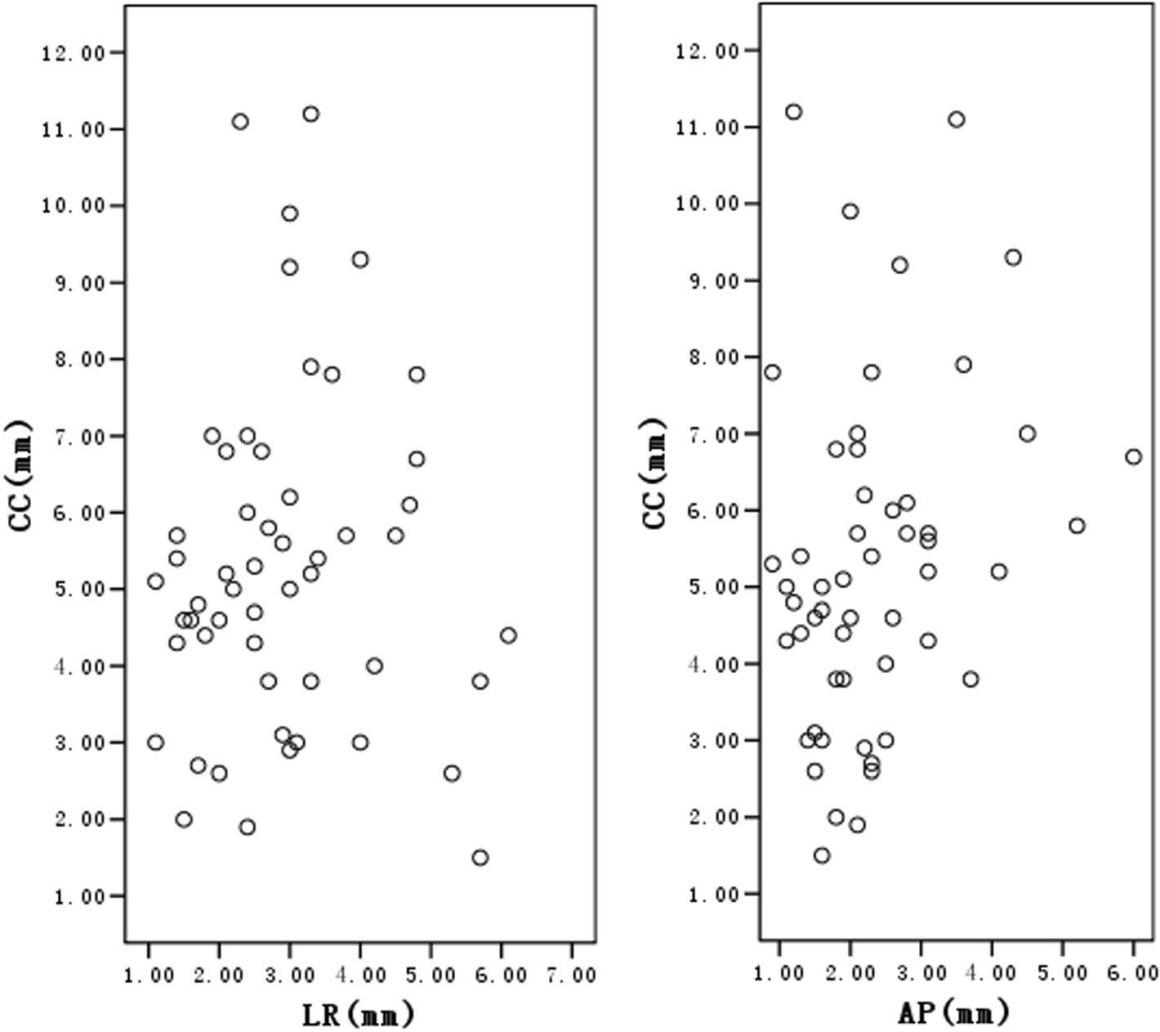
Scatter plots of liver motion in three dimensional directions. Scatter plots illustrating respiration-induced liver motion in the left-right (LR), cranial-caudal (CC), and anterior-posterior (AP) directions for patients undergoing abdominal compression

The mean respiration-induced liver motion for patients undergoing AC in the LR, CC, AP, and 3D vector directions was 2.9 ± 1.2 mm, 5.3 ± 2.2 mm, 2.3 ± 1.1 mm, and 6.7 ± 2.1 mm, respectively. Figure 1 shows scattered plot representations of respiratory liver motion in the LR, CC, and AP directions for patients undergoing AC.

### Predictors of effectiveness of abdominal compression

Table 1 summarizes the association between clinicopathological factors and the effectiveness of AC in the CC direction. Gender, age, BMI, TACE (transarterial chemoembolization) history, liver resection history, tumor area, tumor number, and tumor size (diameter) were analyzed. In univariate comparisons, gender and BMI were significantly associated with the effectiveness of AC in patients with intrahepatic tumors (*p* < 0.05 of both factors). Age (*p* = 0.500), TACE (*p* = 0.669), postoperative recurrence (*p* = 0.659), tumor area (*p* = 0.691), tumor number (*p* = 0.908), and tumor size (diameter) (*p* = 0.934) were not significantly associated with the effectiveness of AC in intrahepatic tumor patients. The two associated factors (gender and BMI) were subsequently used for multivariate analysis.

Table 2 summarizes the association between the effectiveness of AC management in the CC direction and patient gender or BMI, as determined by multivariate analysis. These two factors both remained significant predictors of the likelihood of ineffective AC: gender (*p* = 0.030) and BMI (*p* = 0.006). There was a strong correlation between gender and the effectiveness of AC (odds ratio [OR] = 7.450) and an even stronger correlation between BMI and the effectiveness of AC (OR = 10.842).

### The optimal cut-off value for BMI

**Fig. 2:**
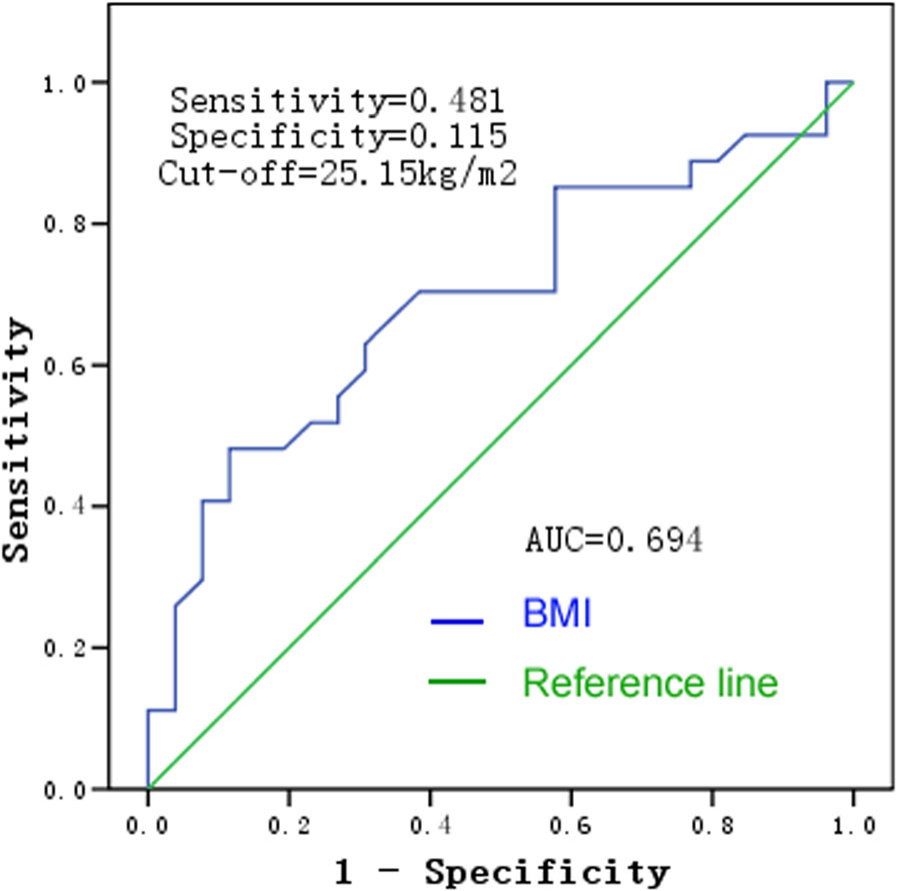
Receiver operating characteristic curve of body mass index (BMI) and breath amplitude in the cranial-caudal direction

The optimal cut-off level of BMI was defined as the BMI with the largest sensitivity and specificity, as determined by receiver operating characteristic (ROC) curve analysis of breath amplitude in the CC direction. The area under the curve (AUC) for BMI was 0.694 (*p* = 0.016) and the optimal cut-off value was 25.15 kg/m^2^, as shown in Fig. 2. When repeating the multivariate logistic regression analysis of the association between BMI and AC effectiveness using the optimal cut-off BMI value of 25.15 kg/m^2^, the *p*-value was 0.006 and 95% CI was 2.012–58.434, which were the same results obtained using the original BMI cut-off value of 25 kg/m^2^.

### Correlation between body mass index and gender

Among the patients who underwent AC, the mean BMI was 22.99 ± 3.92 kg/m^2^ for the females and 23.26 ± 3.44 kg/m^2^ for the males. There was no significant difference between these values (*p* = 0.821). No correlation was detected between BMI and gender. This supports the multivariate analysis findings that BMI and gender were independent factors influencing the effectiveness of AC.

### Respiratory liver motion without abdominal compression

The mean liver respiratory motion in the LR, CC, AP, and 3D vector directions for 46 intrahepatic carcinoma patients in the free-breathing state (without AC) were 3.1 ± 1.3 mm, 9.9 ± 2.6 mm, 2.9 ± 1.4 mm, and 11.0 ± 2.4 mm, respectively. Respiration-induced liver motion was most obvious in the CC direction, ranging from 5.2 to 16.8 mm in these patients who did not undergo AC. The mean liver respiratory motion in the CC direction in the absence of AC was 8.9 ± 2.3 mm for females and 10.4 ± 2.6 mm for males. There was no significant difference between these two values (*p* > 0.05). There was no correlation between free-breathing amplitude in the CC direction and BMI (r = 0.214 and *p* = 0.153 by Pearson correlation analysis).

## Discussion

**Fig. 3:**
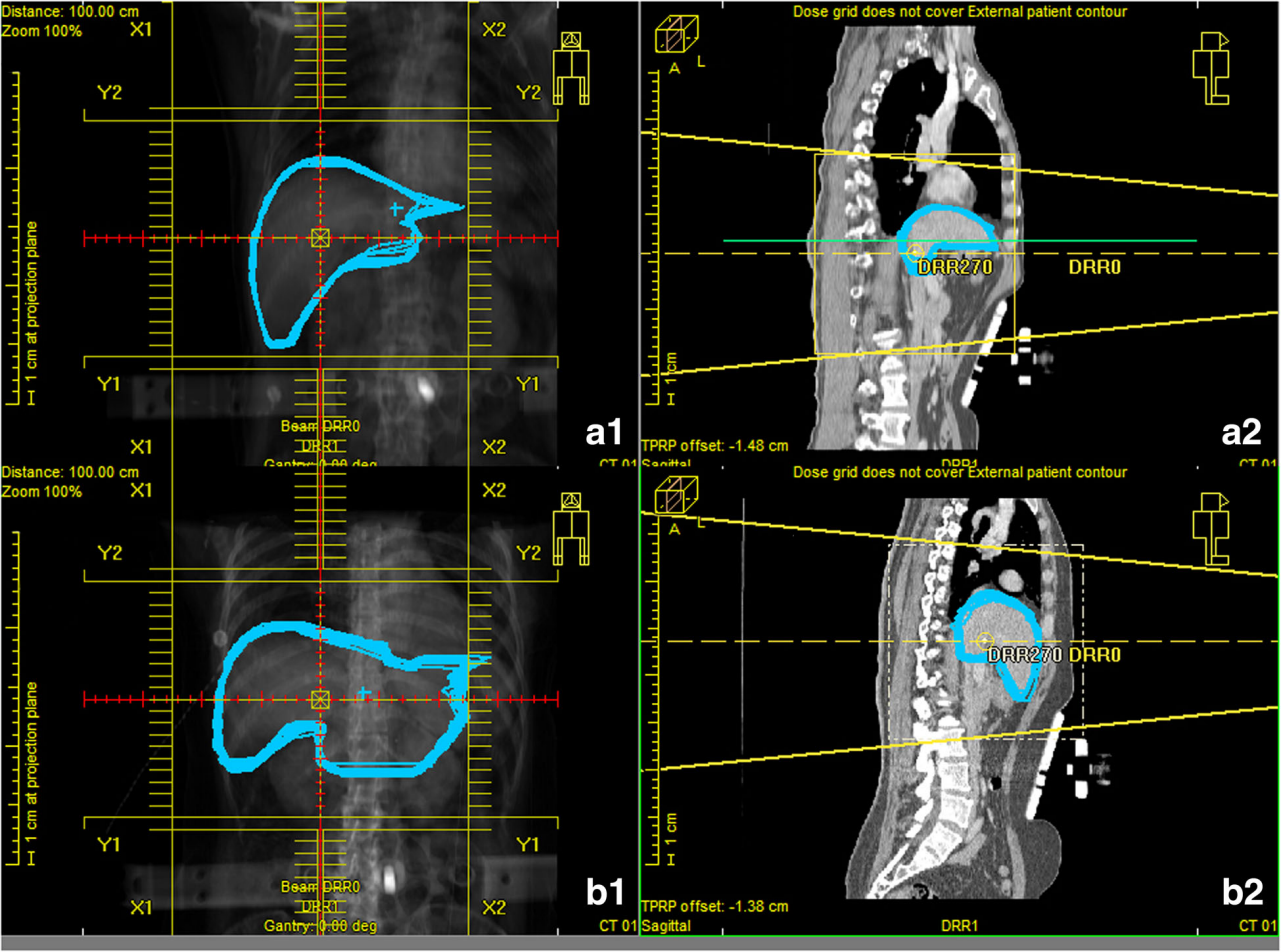
Overlay of 10 liver contours rendered on a digitally reconstructed radiographic image showing the relationship between body mass index and breath amplitude in the 3-dimensional directions from a qualitative perspective. The image in a1 is a tight overlay of 10 liver contours for a patient with a normal body weight (a2), and the image in b1 is a loose overlay for an overweight patient (b2)

In this study, we found that gender and BMI were independent influencing factors associated with the effectiveness of AC. Females had a lower likelihood of AC being ineffective than males. This may be attributable to a more predominant thoracic breathing pattern observed in females. BMI is a tool used to assess weight status based on height, which reflects the amount of body fat to some degree. In this study, no children or athletes were included because their degree of body fat would not be accurately described by the BMI. As shown in Fig. 3, the greater the volume of abdominal adipose tissue depots, the greater the respiration-induced liver motion that would occur when AC was provided. The likely explanation for this finding is that fat accumulating in the abdomen would act as a cushion attenuating the rise in abdominal pressure during AC. Indeed, the waist-height ratio may, at least theoretically, be a more accurate indicator of abdominal obesity than BMI. However, the two parameters (BMI and waist-height ratio) would interfere with each other in multivariate logistic regression analysis, as there would be a correlation between them. At first, we only recorded height and weight values of patients in this study, but not the waistline. We then attempted to measure the waistline of patient using CT image, but found it was not a real waistline for patient under AC because of the compressed abdomen. We chose BMI as the factor evaluated in this study primarily also because it was better known to researchers and readers than the waist-height ratio.

Kitamura et al. [20] reported that tumor location, hepatic cirrhosis, and previous hepatic surgery all had an impact on the intrafractional tumor motion of the liver in the transaxial direction. Tumor motion of patients with liver cirrhosis was significantly larger than that of patients without liver cirrhosis in the LR and AP directions (*p* < 0.004) [20]. We did not evaluate liver cirrhosis as a possibly influencing factor in our study for two main reasons. First, most (70% to 90%) primary liver cancers occurring worldwide are HCC, and most of these tumors arise in patients with liver cirrhosis prior to being diagnosed with HCC [1]. Thus, it is quite likely that the majority of patients in our study had some degree of cirrhosis. Furthermore, there are no diagnostic signs specific for early stage liver cirrhosis according to CT imaging, so we were unable to accurately determine the exact number of patients with liver cirrhosis in this study.

Varying forces on the abdomen may inhibit liver motion to different degrees. For example, using 4D-CT, Heinzerling et al. [10] demonstrated significantly improved control of liver tumor motion with strong AC compared to medium AC. Likewise, varying AC plate positions may inhibit liver motion to different degrees; the further away from the subxiphoid area the compression is applied, the greater the magnitude of liver motion [2]. In the current study, AC was applied during each patient’s end-expiration until maximum tolerability was reached, as indicated by the patient. We found that abdominal breathing clearly switched to thoracic breathing with satisfactory AC, especially in male patients, and forced shallow breathing also occurred [21]. However, forced shallow breathing was difficult to detect in male patients with severe obesity.

Our results suggest that an overweight man undergoing AC may have a high risk of ineffective control of respiration-induced liver motion. Based on our findings, radiation oncologists should predict the effectiveness of AC for patients with intrahepatic tumors by considering their gender and BMI (the independent influencing factors) and chose another respiratory management for patients if they have a high likelihood of the breath amplitude being > 5 mm in the CC direction. However, with current advancements in precision radiotherapy, controlling organ motion continues to be critical for successful treatment in complex cases involving higher doses of radiation. In these instances, it may be more suitable to use a respiratory gating technique to deliver radiation only to the tumor during part of the respiratory cycle [22–24] or active breathing control (ABC), which achieves temporary and reproducible inhibition of respiration-induced motion by monitoring the patient’s breathing cycle and implementing a breath hold at a predefined stage of respiration and air flow direction [25, 26].

Zhao et al. [27] investigated the feasibility and effectiveness of utilizing ABC in 3D-conformal radiation therapy (3D-CRT) for HCC; they concluded that using ABC in 3D-CRT for HCC is feasible and reduces normal liver irradiation. Xi et al. [28] reported that respiratory-gated radiotherapy can further reduce target volumes to spare more surrounding tissue and allow dose escalation, especially for patients with > 1 cm tumor mobility. Cyber Knife [29] should also be considered as a good treatment choice for some patients. Compared with intensity-modulated radiation therapy, helical tomotherapy is one of the techniques for overcoming the effects of respiration during abdominal tumor radiotherapy [30, 31].

Liver deformable registration can be evaluated using MORFEUS, a finite element model (FEM)-based multiorgan deformable image registration method developed by RayStation TPS (RaySearch Laboratories AB, Stockholm, Sweden) [9, 32]. Because of our lack of access to a deformable registration device, we could not use liver deformable registration to enrich our conclusions. Motion artifacts occur frequently in 4D-CT images because of breathing irregularities, which may affect the robustness of measurements. Each patient in the current study underwent basic respiratory training guided by a radiotherapy oncologist and therapist before 4D-CT. The panel “Trigger” of the 4D-CT application software allows visualization of the respiratory waveform, and we were able to observe the respiratory wave immediately prior to the 4D-CT scanning. Although patients were taught to breathe as regularly as possible, we are considering the use of audio-visual feedback to improved breathing regularity in our future clinical research.

## Conclusion

The magnitude of respiration-induced liver motion in patients with intrahepatic carcinoma undergoing AC is affected by gender and BMI. Caution must be taken when trying to reduce respiration-induced liver motion with AC, especially in males and overweight patients with intrahepatic tumors. It may be better for overweight male patients with intrahepatic tumors to select other motion management strategies during external radiotherapy.
